# Caregivers’ treatment-seeking behaviour for children under age five in malaria-endemic areas of rural Myanmar: a cross-sectional study

**DOI:** 10.1186/1475-2875-14-1

**Published:** 2015-01-05

**Authors:** Moe Moe Thandar, Myat Phone Kyaw, Masamine Jimba, Junko Yasuoka

**Affiliations:** Community and Global Health Department, Graduate School of Medicine, the University of Tokyo, Tokyo, Japan; Department of Medical Research (Lower Myanmar), Yangon, Republic of the Union of Myanmar

**Keywords:** Malaria, Treatment-seeking behaviour, Children under age five, Myanmar

## Abstract

**Background:**

A community-based malaria intervention was introduced through fixed and mobile clinics in rural Myanmar. This study attempted to identify treatment-seeking behaviour of caregivers for children under five and the determinants of appropriate treatment-seeking behaviour in mobile clinic villages (MV) and non-mobile clinic villages (NMV) in malaria-endemic rural areas in Myanmar.

**Methods:**

A cross-sectional study was conducted in 23 MV and 25 NMV in Ingapu Township, Myanmar. Appropriate treatment-seeking behaviour was operationally defined as seeking treatment from trained personnel or at a health facility within 24 hours after the onset of fever. Multiple logistic regression analyses were conducted to identify the determinants of appropriate treatment-seeking behaviour.

**Results:**

Among the 597 participants in both types of villages, 166 (35.3%) caregivers sought appropriate treatment. No significant difference in appropriate treatment-seeking behaviour was found between the two types of villages (adjusted odds ratio (AOR), 0.80; 95% confidence interval (CI), 0.51-1.24). Determinants of behaviour include proximity to public health facilities (AOR, 5.86; 95% CI, 3.43-10.02), knowledge of malaria (AOR, 1.90; 95% CI, 1.14-3.17), malaria prevention behaviour (AOR, 1.76; 95% CI, 1.13-2.76), treatment at home (AOR, 0.26; 95% CI, 0.15-0.45), and treatment and transportation costs (AOR, 0.52; 95% CI, 0.33-0.83).

**Conclusions:**

Caregivers’ treatment-seeking behaviour was poor for fever cases among children under age five, and did not differ significantly between MV and NMV. It is necessary to educate caregivers, particularly for early treatment seeking and appropriate use of health care options for fever, and catering to their medical needs. These findings can help promote awareness and prevention, and improve the quality of interventions at the community level.

## Background

Malaria is a major health issue in several tropical and sub-tropical countries [1]. In 2012, there were an estimated 207 million malaria cases and 627,000 malaria deaths, of which 77% of deaths were children under age five [2]. However, malaria mortality has decreased by more than 25% since 2000 due to extensive prevention and control measures [3]. Appropriate malaria treatment administered within 24 hours after the onset of fever can help lower it further [4].

Although malaria is typically treated at health facilities, diagnosis and treatment at community level is effective when access to such facilities is limited. The effectiveness of community-level malaria control measures is influenced by early recognition of symptoms and subsequent treatment-seeking behaviour [5]. It is therefore crucial to obtain region-specific information on treatment-seeking behaviour for malaria, the use of anti-malarial drugs, and obstacles to treatment [6, 7].

Malaria treatment-seeking behaviours are also associated with socio-economic, demographic and personal factors. Other important factors are proximity to health facilities, availability of transportation, knowledge of malaria, a history of malaria, cultural beliefs regarding traditional and herbal medicines, satisfaction with health services, and attitude towards health care providers [1, 8–13].

In Myanmar, malaria is of national concern. Approximately 63% of the country’s population lives in malaria-affected areas. Three major post-natal causes of deaths among one- to 59-months-old children are pneumonia, diarrhoea and malaria [14]. Over the past 20 years, the National Malaria Control Programme (NMCP) has made considerable progress in malaria control. Yet the proportion of under-five children who received anti-malarial drugs was well below 60%, and the proportion of under-five children sleeping under insecticide-treated nets was less than 40% in 2008 [15].

Data on malaria-related treatment-seeking behaviours are limited [12]; the only available data found that self-treatment and seeking assistance from drug vendors were the most common practices in Teikkyi township and Shan Special Region II [12, 16]; and in the China-Myanmar border area, only 32.0% of malaria patients sought treatment within 24 hours and 20.1% were tested for confirming diagnosis [12].

A Myanmar Medical Association-Malaria (MMA-Malaria) project has been implementing a community-based malaria control programme that promotes the rational use of government-recommended anti-malarial drugs and rapid diagnostic tests (RDT). It also introduces fixed and mobile clinics, and recruits village malaria volunteers in malaria-endemic townships for diagnosis and treatment.

However, little is known about treatment-seeking behaviour after introducing this malaria control programme in communities. To evaluate such control programmes, it is critical to obtain baseline information at the initial stages of programme implementation. Therefore, this study attempted to identify caregivers’ treatment-seeking behaviour for children under five and determinants of appropriate treatment-seeking behaviour in mobile clinic villages (MV) and non-mobile clinic villages (NMV) in malaria-endemic rural areas in Myanmar.

## Methods

### Study area

This cross-sectional study was conducted in Ingapu Township, Ayeyarwaddy region, Myanmar. Of 213,064 residents in the township, 22,790 are children under age five. Thirty-one per cent of the total population in Ingapu Township is at high risk for malaria [17]. Malaria morbidity was 16.78 per 1,000 individuals and mortality was 3.75 per 100,000 cases in 2011. The MMA-Malaria project has conducted a malaria control programme through fixed and mobile clinics and village volunteers in this township since 2012. One fixed clinic is located at Kwin Kaut town and 34 malaria-endemic villages were selected for mobile clinic visits based on malaria incidences and accessibility. One volunteer in each selected village was recruited for the period between the mobile clinic visits. The mobile team and volunteers provided malaria diagnosis and treatment, and confirmed malaria patients were provided with malaria-related health education by using pamphlets and long-lasting insecticide nets (LLIN). These activities were gradually expended to 34 villages by June 2013.

### Sampling method

Of the 34 malaria-endemic villages where the MMA-Malaria project intervention was introduced, 23 MV were randomly selected for the study. Out of 225 malaria-endemic villages without MMA-Malaria project intervention, 25 non-intervention villages were selected as a comparison group (NMV), ensuring similarity in residents’ demographics, socio-economic status, geographical location, and proximity to fixed clinics. Villages with rural health centres (RHCs) were excluded. Depending on the population of the village (150–500 residents), approximately 12 participants from each village were selected.

### Participants

Study participants were caregivers of children under five years who had a history of fever during the two months preceding the study. Caregivers were mothers or other family members who were most responsible for attending to their child’s health. Caregivers younger than 18 years and those with mental health problems were excluded. Further, those whose children were ill at the time of the interview were also excluded, as they were likely to seek health care until the child was cured. In high-risk areas, malaria is suspected in every fever case. Therefore, in this study seeking treatment within 24 hours after the fever was set as the indicator of appropriate treatment-seeking behaviour [12, 13].

### Sample size calculation

The sample size was calculated using Epi Info 7 (Centers for Disease Control and Prevention, Atlanta, Georgia). The test’s power was 80% with a 95% confidence interval. Due to limited data about treatment-seeking behaviour for children under five, the prevalence of the dependent variable was estimated to be 70% for this study, based on a previous study in Bago Region in Myanmar [18]. This resulted in a minimum sample size of 246 participants in one group. To compensate for missing responses, at least 300 participants for each group were recruited in this study.

### Measurements and variables

A structured questionnaire was developed based on the WHO Malaria Indicator Survey tool [19] and relevant studies [20–23]. The questionnaire was first developed in English, translated into Myanmar language, and translated back into English by experts on public health and malaria in Myanmar. The questionnaire was pre-tested in a village with comparable proximity and demographic distribution to the selected villages before finalizing the instrument for data collection. The questionnaire contained four major domains: socio-demographic characteristics, knowledge of malaria, malaria prevention behaviour, and malaria treatment-seeking behaviour.

### Outcome variables

The outcome variable was appropriate treatment-seeking behaviour, operationally defined as seeking treatment from trained personnel or at a health facility within 24 hours of the onset of fever [24]. In this study, the first treatment source sought by caregivers was examined if it was trained personnel or health facility. If not, the treatment-seeking behaviour was considered inappropriate.

### Independent variables

Socio-economic and personal factors are known to influence treatment-seeking behaviour. The following were included as independent variables: participants’ age, sex [12], level of education, occupation [1, 9, 10], income [1, 12], proximity to public health services [1], knowledge of malaria [9], prevention behaviour [20] and type of village (MV or NMV).

### Knowledge of malaria

Seven components of knowledge about malaria were measured: symptoms, cause, vulnerable groups, prevention, diagnosis, treatment, and government recommended medication. Each component was assessed by multiple-choice questions, awarded one point for each correct answer. The scores for each component were summed to yield the total knowledge index. The knowledge index scores were categorized into a high and low group, using the median [21, 22].

### Malaria prevention behaviour

Malaria prevention behaviours were measured using a single question with multiple correct choices. Each malaria prevention measure that the participant selected was awarded one point and the total score was summed. The final scores were categorized into a high and low group, using the median [22].

### Data collection

Data were collected in the monsoon season when malaria transmission is high, from August to September 2013. The lead researcher and three trained interviewers conducted face-to-face interviews for about 30 minutes. The interviewers informed caregivers about the purpose and procedure of the research and ensured that confidentiality would be maintained.

In each village, interviewers visited every accessible household to screen them for the study by inquiring whether any children under age five had a history of fever in the previous two months. If there was more than one child with a history of fever in the previous two months, the history of the most recent child was obtained. In total, 302 participants were selected for the survey in MV and 300 participants in NMV.

### Data analysis

After data collection, data were coded and entered using EpiData and analysed with SPSS version 16 (SPSS Inc, Chicago, IL, USA). Of the 602 participants, five were under the age of 18 years, therefore data of 597 caregivers were used. Frequencies and proportions were used for descriptive data. Independent t-tests were used to compare age, number of household members, number of children under five, and income. Chi-square test and Fisher’s exact test were used to compare the differences in proportions. To represent appropriate treatment-seeking behaviour, the time taken to seek treatment and health service provider sought were combined and then categorized into four groups. The first group comprised caregivers who sought treatment at a health facility or from trained personnel within 24 hours. The second comprised caregivers who sought treatment within 24 hours, but not at a health facility or from trained personnel. The third comprised caregivers who sought treatment at a health facility or from trained personnel, but not within 24 hours. The fourth group comprised caregivers who did not seek treatment at a health facility or from trained personnel, nor sought treatment within 24 hours. The first group was termed ‘caregivers with appropriate treatment-seeking behaviour’; the three remaining groups were termed ‘caregivers without appropriate treatment-seeking behaviour’.

To examine appropriate treatment-seeking behaviour, all study participants’ data were combined and two kinds of analyses were performed. First, descriptive analyses were conducted to examine differences in characteristics of caregivers who demonstrated appropriate treatment-seeking behaviour and those who did not. Second, multiple logistic regression was performed to identify the determinants of appropriate treatment-seeking behaviour. Type of village (MV and NMV) was set as an independent variable in multiple logistic regression analysis. Multicollinearity among independent variables was tested before logistic regression. Potential confounders included level of education, occupation and income. All continuous variables were converted to dichotomous variables for multiple logistic regression analysis using the sample median and statistical significance was set at p <0.05.

### Ethical considerations

Ethical approval was obtained from the Research Ethics Committee of the Graduate School of Medicine, the University of Tokyo, Japan, and the Department of Medical Research (Lower Myanmar), Ministry of Health, Yangon, Myanmar. Upon explaining the objectives of the study, written consent was obtained from all respondents and confidentiality was maintained.

Participants’ names were not recorded; instead, identification numbers were used. All the information was treated confidentially and only available to those who directly concerned with this research.

## Results

### Socio-demographic characteristics of participants

In total, 597 caregivers participated in this study (see Table 1). Almost all participants were mothers, married and of Burma ethnicity. Participants’ mean age was 31.5 years (range: 18–75 years). The mean age of children under five with a history of fever during the two months before the interview was 27.4 months (range: 2–59 months); 53.1% of the children were boys.

**Table 1:** **Socio-demographic characteristics of participants**

Variables	MV (n = 299)		NMV (n = 298)		
	n	%	n	%	p value
Number of household members					
≤ 4	183	61.2	183	61.4	0.959
> 4	116	38.8	115	38.6	
Number of children U5 at home					
1	271	90.6	265	88.9	0.491
> 1	28	9.4	33	11.1	
Children’s age (months)					
≤ 27	158	52.8	159	53.4	0.900
> 27	141	47.2	139	46.6	
Children’s sex					
Male	156	52.2	161	54.0	0.650
Female	143	47.8	137	46.0	
Caregivers’ age (years)					
≤ 30	170	56.9	155	52.0	0.235
> 30	129	43.1	143	48.0	
Relationship to child					
Mother	271	90.6	270	90.6	0.989
Other	28	9.4	28	9.4	
Level of education					
Illiterate	22	7.4	21	7.0	0.984
Primary school	209	69.9	208	69.8	
Secondary school or higher	68	22.7	69	23.2	
Occupation					
Farmer	116	38.9	123	41.4	**0.001**
Forest worker	70	23.5	30	10.1	
Other	112	37.6	144	48.5	
Income (US$)					
≤ 60	165	55.9	197	66.1	**0.011**
> 60	130	44.1	101	33.9	
Nearest health service*					
Drug store	172	57.5	160	53.7	**<0.001**
Midwife	68	22.7	62	20.8	
Charlatan/traditional healer	29	9.7	35	11.7	
Malaria volunteer	17	5.7	0	0.0	
Rural health centre	9	3.0	31	10.4	
General practitioner	4	1.3	10	3.4	
Mode of transportation					
On foot	260	87.0	149	83.6	0.241
By vehicle	39	13.0	49	16.4	
Duration					
Within 30 min	291	97.3	287	96.3	0.480
More than 30 min	8	2.7	11	3.7	

Notes: MV: Mobile clinic villages; NMV: non-mobile clinic villages; children U5: children under five; Chi-square test p-vale (*Fisher’s exact test).

No significant differences between MV and NMV were observed for socio-demographic characteristics, except for occupation, income and nearest health service provider. In MV, 23.5% of residents were forest workers, while 10.1% were forest workers in NMV (p = 0.001); 44.1% in MV as opposed to 33.9% in NMV had a monthly income of more than 60US$ (p = 0.011). In both types of villages, the closest health service providers were drug stores (MV 57.5% *vs* NMV 53.7%), followed by midwives (MV 22.7% *vs* NMV 20.8%).

### Caregivers’ treatment-seeking behaviour

In more than 80% of the households mothers were the decision-makers for seeking treatment outside the home in both types of villages (see Table 2). More than 50% of the caregivers did not medicate at home before seeking treatment, and more than 66% sought treatment outside the home in both types of villages. About 90% of the caregivers raised “disease severity” as the most important deciding factor to seek treatment. In both types of villages, more than 50% of the caregivers sought treatment within 24 hours of the onset of fever and was comparative across MV (57%) and NMV (62%).

**Table 2:** **Caregivers’ treatment-seeking behaviour**

Variables	MV (n = 299)		NMV (n = 298)		
	n	%	n	%	p value
Decision-maker for seeking treatment					
Mother	246	82.3	251	84.2	0.523
Other	53	17.7	47	15.8	
Deciding factor					
Disease severity	272	91.3	265	88.9	0.337
Other	26	8.7	33	11.1	
Treatment at home					
Yes	127	42.5	115	38.6	0.334
No	172	57.5	183	61.4	
Treatment outside					
Yes	229	76.6	241	80.9	0.201
No	70	5.8	57	19.1	
Reason for not receiving treatment					
Quick recovery	47	67.1	44	77.2	0.211
Other	23	32.9	13	22.8	
Time taken to seek treatment					
Within 24 hours	131	57.2	150	62.2	0.441
Other	98	42.8	91	37.8	
Primary health service provider*					
Midwife	73	31.9	58	24.1	**0.041**
GP	62	27.1	62	25.7	
Drug store	55	24.0	65	27.0	
Charlatan/traditional healer	15	6.6	20	8.3	
RHC	14	6.1	31	12.9	
Malaria volunteer/mobile clinic	6	2.6	1	0.4	
Hospital	4	1.7	4	1.7	
Reason for choosing primary health service					
Trust	111	48.5	117	48.5	0.097
Proximity	64	27.9	54	22.4	
Other	28	12.3	41	17.0	
Famous	10	4.4	5	2.1	
Inexpensive	16	7.0	24	10.0	
Mode of transportation					
On foot	108	47.2	119	49.4	0.807
Motorbike	68	29.7	72	29.9	
Other	53	23.1	50	20.7	
Blood test					
Yes	17	7.4	16	6.6	0.739
No	212	92.6	225	93.4	
Location where blood test was conducted*					
Midwife	5	29.4	6	37.5	0.230
GP	7	41.2	3	18.8	
Hospital/RHC	2	11.8	6	37.5	
Malaria volunteer/mobile clinic	3	17.6	1	6.3	
Health education conducted with patient					
Drug timetable	7	87.5	5	100.0	0.411
Drug compliance	7	87.5	5	100.0	0.411
Preparedness for worsened symptoms*	6	75.0	2	40.0	0.293
Follow up*	2	25.0	2	40.0	1.000
Severe malarial symptoms*	2	25.0	1	20.0	1.000
Side effects*	1	12.5	0	0.0	1.000
Total cost (US$)					
≤ 1.1	111.0	48.5	125	51.9	0.462
> 1.1	118.0	51.5	116	48.1	
Used MMA service					
Yes	52	17.4	32	10.7	**0.019**
No	247	82.6	266	89.3	

Notes: MV: Mobile clinic villages; NMV: non-mobile clinic villages; GP: general practitioner; RHC: rural health centre; MMA: Myanmar Medical Association; Chi-square test p-vale (*Fisher’s exact test).

The most frequented primary health service provider was the midwife (31.9%) for caregivers who sought treatment outside the home in MV (n = 229), whereas in NMV (n = 241), the drug stores were the most frequented primary source for treatment (27.0%) (p = 0.041). Only 2.6% of caregivers sought treatment from mobile clinics or malaria volunteers in MV. Further, 49.3% of caregivers took longer than 15 minutes to reach the first source for treatment in MV as opposed to 39.0% in NMV (p = 0.024).

In MV, of the total 229 children who received treatment outside their homes, 17 were tested for malaria; 15 (6.6%) children received a malaria diagnosis using blood tests with RDT, and two children (0.9%) received a diagnosis using microscopy. In NMV, of the total 241 children who received treatment outside home, 16 children were tested for malaria; blood test with RDT were conducted on 15 children (6.2%) and one child (0.4%) received a diagnosis with microscopy. In MV, blood tests were conducted most frequently at general practitioners’ (GPs) clinics, while in NMV, public health facilities (midwives, hospitals, and RHCs) were most frequently visited for blood tests. Only five children in MV and three in NMV received positive results for malaria on the blood test.

In both types of villages, 88 caregivers consulted more than one health service provider and 20 caregivers consulted three providers. When mobile clinics and malaria volunteers were sought, they were always the first-choice health service provider (data not shown in Table). Fifty-two caregivers (17.4%) from MV and 32 caregivers (10.7%) from NMV had sought MMA mobile clinics or malaria volunteers at least once (p = 0.019).

### Caregivers’ knowledge of malaria

In both types of villages, more than 85% of the caregivers stated chills and rigor as symptoms of malaria (see Table 3). More than 60% in both types of villages included sweating as a symptom of malaria. About 50% of caregivers in both types of villages were aware that children under five were especially vulnerable to malaria infection and more than 90% knew that mosquito bites cause malaria.

**Table 3:** **Caregivers’ knowledge of malaria**

Variables	MV (n = 299)		NMV (n = 298)		
	n	%	n	%	p value
Symptoms					
Fever (yes)	270	95.4	263	97.4	0.208
Chills and rigors (yes)	248	87.6	255	94.4	**0.005**
Headache (yes)	211	74.6	215	79.6	0.156
Sweating (yes)	177	62.5	202	74.8	**0.002**
Vulnerable groups					
Under-five children (yes)	162	57.2	132	48.9	**0.049**
Pregnant mothers (yes)	139	49.1	116	43.0	0.147
Forest workers (yes)	256	90.5	248	91.9	0.565
Farmers (yes)	170	60.1	150	55.6	0.282
Causes					
Mosquito bite (yes)	279	98.6	256	94.8	**0.012**
Coughing and sneezing (no)	46	16.3	51	18.9	0.415
Contact (no)	83	29.3	81	30.0	0.863
Drinking (no)	43	15.2	37	13.7	0.618
Bathing (no)	63	22.3	48	17.8	0.188
Eating bananas (no)	74	26.1	63	23.3	0.443
Prevention method					
Use mosquito/bed net (yes)	245	86.6	237	87.8	0.672
Use LLIN (yes)	239	84.5	228	84.4	0.998
Avoid mosquito bites (yes)	235	83.0	234	86.7	0.235
Use mosquito coil (yes)	196	69.3	202	74.8	0.146
Use mosquito repellent (yes)	158	55.8	139	51.5	0.305
Wear long-sleeved clothing (yes)	219	77.4	209	77.4	0.995
Clean environment (yes)	247	87.3	235	87.0	0.932
Cover water containers (yes)	241	85.2	235	87.0	0.524
Diagnosis					
Blood test (yes)	225	79.5	220	81.5	0.558
Fever with chills and rigor (no)	196	37.5	114	42.2	0.252
Observation (no)	26	9.2	22	8.1	0.664
Curable drugs					
Anti-malarials (yes)	169	91.4	165	91.2	0.948
Antibiotics (no)	94	50.8	89	49.2	0.754
Traditional medicine (no)	64	34.6	70	38.7	0.418
Vitamins (no)	83	44.9	99	54.7	0.060
Government recommended drug (Coartem)	44	55.0	47	75.8	**0.010**

Notes: MV: Mobile clinic villages; VWOMC: non-mobile clinic villages; LLIN: long-lasting insecticide nets; Chi-square test p-vale.

In both types of villages, more than 80% of caregivers answered malaria could be prevented by using mosquito nets or LLIN. Nearly 80% of respondents in both types of villages answered that malaria could be diagnosed by blood tests and over 90% knew that malaria could be treated using anti-malarial drugs. Moreover, 55.0% of caregivers in MV compared to 75.8% in NMV could provide the name of the recommended drug (p = 0.010).

### Caregivers’ malaria prevention behaviour

In both types of villages, the most popular method for malaria prevention, with nearly 100% response rate, was using bed nets (see Table 4). This was followed by avoiding mosquito bites (more than 92%), and wearing long-sleeved shirts and trousers (more than 71%) in both villages. The use of LLIN was low in both types of villages (less than 15%).

**Table 4:** **Caregivers’ malaria prevention behaviour**

Variables	MV (n = 299)		NMV (n = 298)		
	N	%	n	%	p value
Preventive action					
Using mosquito/bed net	278	98.2	270	100.0	**0.028**
Avoid mosquito bites	262	92.6	256	94.8	0.281
Wearing long-sleeved clothing	221	78.1	194	71.9	0.090
Using mosquito coil	160	56.5	137	50.7	0.172
Using LLIN	34	12.0	39	14.4	0.399
Using mosquito repellent	10	3.5	14	5.2	0.341

Notes: MV: Mobile clinic villages; NMV: non-mobile clinic villages; LLIN: long-lasting insecticide nets; Chi-square test p-vale.

### Determinants of appropriate treatment-seeking behaviour

Table 5 shows the determinants of appropriate treatment-seeking behaviour. Proximity to public health facilities was positively associated with appropriate treatment-seeking behaviour (adjusted odds ratio (AOR), 5.86; confidence interval (CI), 3.43-10.02). Caregivers who gave their child any medicine at home before seeking treatment outside were less likely to seek appropriate treatment (AOR, 0.26; CI, 0.15-0.45). Caregivers who spent less on treatment and transportation to the nearest health services were less likely to seek appropriate treatment (AOR = 0.52; CI 0.33-0.83). Compared to caregivers who had low levels of knowledge, caregivers with high levels of knowledge were more likely to seek appropriate treatment (AOR = 1.90; CI 1.14-3.17). Similarly, caregivers who had high levels of malaria prevention behaviour were more likely to seek appropriate treatment (AOR = 1.76; CI 1.13-2.76).

**Table 5:** **Determinants of appropriate treatment-seeking behaviour**

Variables	AOR	(95% CI)
Type of village		
NMV (ref.)		
MV	0.80	(0.51-1.24)
Number of children U5 at home		
> 1 (ref.)		
1	1.05	(0.48-2.30)
Children’s age (months)		
≤ 27 (ref.)		
> 27	0.70	(0.44-1.09)
Children’s sex		
Female (ref.)		
Male	1.26	(0.81-1.93)
Caregivers’ age (years)		
≤ 30 (ref.)		
> 30	0.75	(0.47-1.19)
Marital status		
Other (ref.)		
Married	1.70	(0.53-5.45)
Level of education		
Illiterate (ref.)		
Primary school	0.99	(0.38-2.61)
Secondary school or higher	1.06	(0.37-3.05)
Occupation		
Other (ref)		
Farmer	0.87	(0.54-1.41)
Forest worker	0.80	(0.42-1.51)
Income (US$)		
≤ 60 (ref.)		
> 60	1.04	(0.65-1.66)
Nearest health service		
**Inappropriate health service (ref.)		
Public health service	5.86	(3.43-10.02)*
Private health service	1.69	(0.52-5.46)
Mode of transportation		
By vehicle (ref.)	1.84	(0.94-3.61)
On foot		
Proximity to nearest health service		
More than 30 min (ref.)		
Within 30 min	3.42	(0.82-14.16)
Treatment at home		
No (ref.)		
Yes	0.26	(0.15-0.45)*
Total cost (US$)		
> 1.1 (ref)		
≤ 1.1	0.52	(0.33-0.83)*
Knowledge level		
Low (ref.)		
High	1.90	(1.14-3.17)*
Preventive action		
Low (ref.)		
High	1.76	(1.13-2.76)*

Notes: MV: Mobile clinic villages; NMV: non-mobile clinic villages; Children U5: children under five; AOR: adjusted odds ratio; CI: Confidence interval; *p value <0.05; **Inappropriate health service includes drug stores, charlatans and traditional healers.

## Discussion

This study revealed several important findings regarding caregivers’ treatment-seeking behaviour for children under age five in malaria endemic areas of rural Myanmar. First, caregivers’ treatment-seeking behaviour was poor; only one-third demonstrated appropriate treatment-seeking behaviour, and the rates of appropriate treatment-seeking behaviour did not differ significantly between MV and NMV. Caregivers’ knowledge of malaria, malaria prevention behaviour and proximity to public health service were important determinants of appropriate treatment-seeking behaviour. At the same time, treatment at home and total cost for treatment and transportation were negatively associated with appropriate treatment-seeking behaviour. Midwives played an important role in treatment-seeking behaviour, as most caregivers first approached the local midwife for their child’s illnesses.

Only about one-third of caregivers demonstrated appropriate treatment-seeking behaviour. Approximately 20% of the children with fever were not taken to any health services for advice or treatment. This situation appears to be much worse than reported in a study conducted in Wa region in Myanmar, wherein 12.5% of caregivers did not seek treatment for their child’s fever [12]. Despite this, the present study found that participants’ treatment-seeking behaviour was more favourable than those in several study sites in other countries [7, 25–27].

The delay in seeking treatment and the decision to utilize untrained health services are a grievous concern as only about one-third of the caregivers sought treatment for their children at a health facility or from trained personnel within 24 hours of the onset of fever. Another third had consulted with trained personnel or at health facilities but only after 24 hours. A quarter of all caregivers sought treatment within 24 hours, but from untrained health services including drug stores, charlatans and traditional healers; 8.5% sought treatment at untrained health services after 24 hours. A study conducted in India showed that children under five were at high risk because they had the least timely and least effective treatment for febrile illnesses among all age groups [5].

In this preliminary survey to determine baseline rates, the presence of mobile clinics (MV) was not associated with appropriate treatment-seeking behaviour for children under five. This may be explained by the limited activities conducted by the mobile team and volunteers for the residents of the target villages in the initial stages of intervention. The initial stage included malaria diagnosis for fever patients and treatment specifically for confirmed malaria cases visiting the clinic, wherein only the patients benefitted from their activities that were not extended to meet community needs. Further, circumstances between MV and NMV were not identical despite efforts to match both. That is, MV were selected because of their relatively inaccessible location (remote, bordering forests where malaria vectors breed, and at a great distance from RHCs). The caregivers were inadvertently more familiar with inappropriate health services, such as drug stores, than mobile clinics and village volunteers in rural areas. However, previous studies in Bago Region in Myanmar concluded that having volunteers specifically trained for implementing malaria control programmes can improve accessibility and administration of health care in villages without health staff, although overall they may remain low [18].

One of the behaviours that led to the delay in seeking treatment was treatment at home; almost 50% of the children with fever were treated at home before seeking treatment outside. Individuals are more likely to begin with self-medication at home to minimize both expenditure and the burden of reaching a facility in remote areas where transportation and health facilities are scarce [28–30]. A high proportion of fever cases were first treated at home with shop-bought drugs before visiting health facilities [31]. Caregivers who administered medication at home were not likely to seek appropriate treatment [12], possibly because the child recovered after self-medication and/or other first-aid measures, such as tepid sponging.

Greater awareness about malaria and undertaking a broader range of preventive actions for malaria influence appropriate treatment-seeking behaviour. A study in Cambodia showed that early recognition of malaria symptoms is the first important step to treatment seeking [20]. In the present study, although caregivers were aware of malaria symptoms, about 50% were unaware that children under five and pregnant mothers are especially vulnerable to malaria. A previous study in Tikekyi township,Yangon region and four townships in Bago region, Myanmar demonstrated that the level of awareness about malaria was low compared to the average score used in the studies [16, 32].

The most popular health service providers in this study were midwives, primarily because caregivers’ trust them being qualified and experienced health providers [33, 34]. Another reason is that midwives have served villagers for longer than the village malaria volunteers [18]. A survey conducted by Myanmar Artemisinin Resistance Containment (MARC) showed that the public sector, including RHCs and midwives, was cited as the most popular source for treatment of malaria [35]. Drug stores also played an important role in the present study, as one-quarter of the caregivers sought treatment from them. A similar situation was observed in sub-Saharan Africa [36]. Proximity to drug stores may have encouraged individuals to use them to save on transportation [37].

Findings from this study should be considered in the context of some limitations. First, caregivers were asked about the fever of their children under five during the previous two months, thus responses might reflect recall bias. Nevertheless, the items utilized in data collection were drawn from validated and reliable instruments that have been used in a variety of settings. Second, this study was unable to explore causal relationships because of a cross-sectional study design.

Despite these limitations, this study is valuable as it identified the determinants of caregivers’ treatment-seeking behaviour for children under five presenting with fever in malaria-endemic, rural Myanmar. This study provides baseline findings for the initial stage of the implemented intervention.

## Conclusions

Caregivers’ treatment-seeking behaviour was poor for their children under five with fever, as only one-third demonstrated appropriate treatment-seeking behaviour. Further, baseline treatment-seeking behaviour for fever cases did not differ significantly between MV and NMV. Caregivers’ knowledge of malaria, malaria prevention behaviour and proximity to public health services were important determinants of appropriate treatment-seeking behaviour. At the same time, treatment at home and total cost for treatment and transportation were negatively associated with appropriate treatment-seeking behaviour. The role of the midwife was important, as most caregivers first sought their assistance for their children’s illnesses.

Greater awareness and health education for caregivers are necessary, particularly on early treatment-seeking and appropriate use of health care options for fever. These findings will be utilized to improve the quality of the intervention and will be compared with follow-up data collected at a later stage to evaluate its effectiveness at the community level.
